# Neuroprotection of reduced thyroid hormone with increased estrogen and progestogen in postpartum depression

**DOI:** 10.1042/BSR20182382

**Published:** 2019-09-03

**Authors:** Dan Li, Yangyao Li, Yun Chen, Haiyan Li, Yuqi She, Xialan Zhang, Shuang Chen, Wanying Chen, Guodong Qiu, Haiqing Huang, Shuyao Zhang

**Affiliations:** 1 Gynaecology and Obstetrics, The Second Affiliated Hospital of Shantou University Medical College, Shantou 515031, P.R. China; 2 Department of Pharmacy, Cancer Hospital of Shantou University Medical College, Shantou 515031, P.R. China; 3 Department of Pharmacy, Guangzhou Red Cross Hospital Affiliated of Ji-Nan University Medical College, Guangzhou 510220, P.R. China; 4 Department of Nursing, Guangzhou Red Cross Hospital Affiliated of Ji-Nan University Medical College, Guangzhou 510220, P.R. China; 5 Department of Ultrasound, Cancer Hospital of Shantou University Medical College, Shantou 515031, P.R. China

## Abstract

**Background:** Postpartum depression (PPD) is a common serious mental health problem. Recent studies have demonstrated that hormone therapy serves as a promising therapeutic approach in managing PPD. The present study aims at exploring the role of thyroid hormone (TH), estrogen and progestogen in patients with PPD.

**Methods:** Initially, PPD patients were enrolled and a PPD mouse model was established. The serum levels of estradiol (E2), progesterone (P), triiodothyronine (T3), thyroxine (T4), free triiodothyronine (FT3), free thyroxine (FT4), and thyroid-stimulating hormone (TSH) were subsequently measured. Next, in order to identify the effects of TH, estrogen and progestogen on PPD progression, mice were administrated with E2, P, contraceptives (CA), Euthyrox and methimazole (MMI). Besides, the body weight, activities, basolateral amygdala (BLA) neuron cell structure and the related gene expression of mice were analyzed.

**Results:** The PPD patients and the mice showed elevated serum levels of T3, T4, FT3 and FT4 along with diminished E2, P and TSH levels. In the mice administered with a combination of E2, P, and MMI, decreased TH and increased estrogen and progestogen were detected, which resulted in increased body weight, normal activities, and BLA neuron cell structure. Moreover, brain-derived neurotrophic factor (BDNF) and cAMP-responsive element-binding protein (CREB) were both up-regulated in PPD mice administrated with a combination of E2, P, and MMI, which was accompanied by decreased TH and elevated estrogen and progestogen.

**Conclusion:** Taken together, reduced TH combined with enhanced estrogen and progestogen confers neuroprotection in PPD, highlighting a potential target in prevention and treatment of PPD.

## Introduction

The first week after giving birth is recognized as a critical period for mothers, who may present with lactation failure and postpartum depression (PPD) [1]. As a common complication of childbearing, PPD is estimated to affect 13–19% of all mothers after delivery [2]. Mothers of preterm and low-birth-weight infants have been reported to carry a significantly higher risk for depression than mothers of term infants after parturition [3]. PPD also exert adverse effects on children’s development since children of depressive mothers are more prone to succumbing to mental disorders in their future life [4]. Pertinent risk factors associated with PPD include a history of previous PPD or affective disorders, previous stressful life events, lack of social support, low self-esteem and negative early breastfeeding experiences [5]. Women suffering PPD often exhibit certain behaviors of anxiety and depression (both as affective states and as clinical disorders) [6].

Estrogen has been highlighted with the capacity of mitigating behaviors induced by anxiety and depression after delivery by mediating estrogen receptor α [7]. Moreover, estrogen has been suggested to regulate transcription via cross-talk with intracellular estrogen receptors in target tissues, such as ESR1 and ESR2, thus playing a role in central nervous system signaling pathways [8]. It has been well established that severe depression occurs as a result of the loss of hippocampal volume, which is caused by hippocampal neuron death [9]. A close link has been identified among estrogen, mitochondrial function, neuronal survival, cognition and neuroinflammation via both genomic and non-genomic signaling pathways [10]. In addition, progestogen has also been suggested as a potential PPD treatment due to its neuroprotective and anti-inflammatory roles in central nervous system activity at physiological concentrations [11,12]. Thyroid dysfunction is associated with the physiological changes after delivery due to thyroid autoimmunity in some cases, thus acting as a marker for PPD [13]. Evidence has been reported that thyroid hormone (TH) may increase the risk of PPD due to its abnormal expression during the early postnatal period which has been linked with severe neurological deficits [14,15]. The concentrations of free triiodothyronine (FT3) and free thyroxine (FT4) share a significant correlation with depression severity and exhibiting a notable impact on the clinical outcomes of patients suffering from major depressive disorder [16]. The aforementioned three hormones have all been reported to exert certain effects on the progression of PPD, while the finer mechanisms of their role in PPD remain poorly understood. Herein, the present study aims to identify the influences of the administration of TH, estrogen and progestogen on PPD, which might shed new lights for prevention and treatment of PPD.

## Materials and methods

### Study subjects

A total of 58 parturient women hospitalized at maternity ward of the Second Affiliated Hospital of Shantou University Medical College from December 2015 to August 2017 were enrolled in the present study and regarded as the observation group. Among the enrolled patients, the median age was 28.95 ± 4.24 years, ranging from 24 to 39 years, with a median gestational period of 39.00 ± 0.76 weeks, ranging from 38 to 41 weeks. Based on the diagnostic criteria for PPD of American Psychiatric Association Diagnostic and Statistical Manual of Mental Disorders (DSM-IV) [17], the enrolled patients were diagnosed with PPD when their respective Edinburgh Postnatal Depression Scale (EPDS) scores were greater than 13 [18]. All participants provided a complete medical record. These patients who had a history of depression during pregnancy, and suffered from mental retardation and concomitant major organ diseases were excluded. In addition, 60 healthy parturient women hospitalized at the Second Affiliated Hospital of Shantou University Medical College during the same period were enrolled as the control group. The median age was 28.66 ± 4.58 years, ranging from 22 to 38 years, while the median gestational period was 38.91 ± 1.11 weeks, ranging from 36 to 42 weeks.

A total of 112 C57BL/6 female mice (aged 6–8 weeks, weighing 18–22 g with the median weight of 19.88 ± 1.45 g) were included in the present study. The mice were fed with standard forage and granted free access to food and water at 24 ± 2°C with humidity of 50–60% and a 12-h light cycle with lights on at 8:00. Next, 96 mice were selected for PPD model establishment and the remaining 16 mice were regarded as the normal group.

### Detection of serum hormone levels in postpartum women

At the 3rd day after delivery, 5 ml venous blood was collected from subjects who were on an empty stomach in the two groups in the morning, centrifuged in a centrifugal machine for blood plasma separation, and stored at −80°C. Electrochemiluminescence immunoassay (ECLIA) was used to evaluate the serum levels of estradiol (E2), progesterone (P), triiodothyronine (T3), thyroxine (T4), FT3, FT4, and thyroid-stimulating hormone (TSH) based on the instructions of hormone detection kits (Roche Diagnostics Ltd., Penzberg, Germany).

### Model establishment [19]

The mice were anesthetized using 1% pentobarbital sodium at 35 mg/kg, fixed on the operation table in a supine position, and shaved accordingly. After the mice had been subjected to conventional disinfection, two incisions (0.5–1.0 cm) were made under aseptic conditions in the region 1 cm below the costal margin of abdomen back and at 0.5 cm of both sides of the spine. The skin and muscle of the mice were separated by blunt dissection, followed by bilateral ovariectomy. Finally, the incisions were closed in layers after observation of bleeding. At the 3rd day, the muscle was injected with penicillin (300000 units) for infection prophylaxis.

On the 8th day (the 1st day of model establishment) post ovariectomy, the mice were subcutaneously injected with 0.1 ml of the mixture containing 0.5 μg E2 and 0.8 mg P at 9: 00 am. At the 17th to 23rd day, mice underwent subcutaneous injection with 0.1 ml E2 (10 μg) every day, which was stopped on the 24th day. E2 and P were prepared with olive oil. The normal mice were subcutaneously injected with 0.1 ml olive oil.

### Animal grouping and treatment

The 112 mice were assigned into the normal (normal mice administrated with normal saline), PPD (PPD mice administrated with normal saline), E2 + P (PPD mice intraperitoneally injected with 0.4 mg/ml E2 and 0.4% P), contraceptives (CA; PPD mice administrated intragastrically with the mixture of 1.5 ml desogestrel and ethinylestradiol and 50 ml normal saline), Euthyrox (PPD mice administrated intragastrically with a mixture of 6 μg levothyroxine sodium and tri-distilled water), methimazole (MMI; PPD mice administrated intragastrically with 0.6 mg/ml per day of MMI), and E2 + P + MMI (administration with of E2, P and MMI) groups.

### Determination of serum hormone levels in mice

Three mice were selected from the normal and the PPD groups respectively. After a 1 week period of gestation, the mice were injected with 0.2 ml 0.5% Evans blue via the tail vein, after which their eyeballs were extracted, with approximately 1.5 ml blood collected and placed into a 2 ml eppendorf (EP) tubes. Next, the serum was separated after centrifugation at 1610×***g***, while the serum levels of E2, P, T3, T4, FT3, FT4 and TSH were determined accordingly.

### Detection of body weight

On the 1st, 11th and 23rd day post model establishment, eight mice from each group were weighed, respectively.

### Sucrose preference test (SPT) [19]

After the mice had been granted free access to water for 2 day, the water was replaced with two bottles of 1% sucrose solution. Two days later, the mice were deprived of water for 1 day. Afterwards, the mice were given the choice to drink from the quantified drinking water and sucrose solution for 1 day, after which the bottles were removed. Next, water and sucrose solution consumption was calculated. The sucrose preference was regarded as the percentage of consumed sucrose solution relative to the total amount of liquid intake.

### Open field test [20]

An open field test (OFT) box (100 cm × 100 cm × 40 cm) was used in a noise-controlled room with the underside and inner wall were made black. The broad field base was divided into 25 parts using red lines. The mice were settled in the central small square at the bottom, with a 5-min period of free exploration permitted, including horizontal and vertical activities in addition to bladder and bowel movements recorded.

### Forced swimming test (FST) [21]

The apparatus for the forced swimming test (FST) used was a cylinder (15 cm diameter × 25 cm height) filled with water (15 cm depth at 25 ± 3°C). The FST day 1, animals were placed into the water of the cylinder for 10 min. The mice were subsequently returned to the cage. On the next day, the mice were forced to swim for 6 min under the same conditions as above. The immobility time during the final 5 min of the testing period was then recorded.

### Tail suspension test (TST) [19]

The distal end (1.5 cm) of the mice’s tail was suspended in a white resin stripping box (40 cm × 40 cm × 40 cm) which was placed at 15 cm from the ground. The immobility time was recorded during the final 5 min within a 6 min testing period.

### Elevated plus-maze (EPM) test [22]

The apparatus comprises two 25 cm × 8 cm × 0.5 cm open arms and two 25 cm × 8 cm × 12 cm closed arms whose top was open that extended from the common central platform (8 cm × 12 cm). The apparatus was elevated to a height of 50 cm above the floor and placed in a condition without external disturbance. During the experiments, the mice were settled in the center of the elevated plus-maze (EPM), with their heads facing the open arms, after which the curtain was drawn. The experimenters were required to be performed at a distance of 1 m away from the EPM, with the monitor then turned on and used to record the number of times of mice entering the open and closed arms over a 5 min period. Next, the mice were returned to their respective cages, after which the EPM was cleaned and wiped using 75% alcohol to eliminate any potential smell that could potentially affect the mice in the subsequent experiments. The percentage of the times of mice entering the open arms = the times of mice entering the open arms/(the times of mice entering the open arms + the times of mice entering the closed arms). The percentage of time spent in the open arms = the time spent in the open arms/(the time spent in the open arms + the time spent in the closed arms).

### Hematoxylin–eosin staining

Three mice were collected from each group, anesthetized with 1% pentobarbital sodium at 35 mg/kg and killed by cervical dislocation. After the brains had been collected, the cortical vessels were completely peeled off, fixed in 4% polyoxymethylene, conventionally embedded in paraffin and sectioned. The sections were then dewaxed using xylene, dehydrated with gradient alcohol, stained with hematoxylin, differentiated with hydrochloric acid–ethanol, counterstained in eosin, hydrated and mounted. The sections were then analyzed under a microscope.

### Observation of basolateral amygdala structure

Three mice were selected from each group, anesthetized by an intraperitoneal injection with 1% pentobarbital sodium at 35 mg/kg. After the brain tissues had been exposed, the lateral basolateral amygdala (BLA) was isolated, fixed with 2.5% glutaraldehyde and then immersed in ethanol and acetone. The purified acetone and embedded solution were then mixed, allowed to stand at room temperature overnight, and then placed in a 37°C incubator overnight. The tissues were then sliced into 70-nm sections. After staining, the microstructure of BLA was photographed and observed under a transmission electron microscope (TEM, JEM-2000EX, JEOL, Tokyo, Japan). Synapse density was analyzed using the body length frequency method, with ImageJ analysis software utilized for statistical analysis.

### Reverse transcription quantitative polymerase chain reaction

A total of 100 mg BLA tissue samples were treated with diethypyrocarbonate (DEPC), washed under tap water, and placed into EP tubes. The total RNA was then extracted using an RNA extraction kit (Invitrogen Inc., Carlsbad, CA, U.S.A.) and reversely transcribed into cDNA using a reaction system consisting of 20 μl based on the instructions of PrimeScript RT reagent kit. The reaction conditions were performed as follows: 37°C for 30 min and 98°C for 5 min. Glyceraldehyde-3-phosphate dehydrogenase (GAPDH) was regarded as the internal reference, with product amplification performed accordingly. After the products had been centrifuged, they were allowed to react in a PCR instrument under the following reaction conditions: pre-denaturation at 95°C for 5 min, and 32 cycles of denaturation at 94°C for 30 s, annealing at 55°C for 30 s and extension at 72°C for 90 s. The primers employed are listed in Table 1, which were designed and synthesized by Takara Biotechnology Inc. (Dalian, Liaoning, China). The fold changes were calculated with the relative quantification 2^−△△*C*_t_^ method. The experiment was repeated three times independently.

**Table 1:** Primer sequences for RT-qPCR

Primer	Sequence
GAPDH	F: 5′-CCCTTCATTGACCTCAACTAC-3′
	R: 5′-CCACGACTCATACAGCACC-3′
BDNF	F: 5′-GCCTCCTCTACTCTTTCTGC-3′
	R: 5′-ATGGGATTACACTTGGTCTC-3′
CREB	F: 5′-TAGTCCCAGCAACCAAGT-3′
	R: 5′-GGACGCCATAACAACTCCAG-3′

Notes: F, forward; R, reverse.

### Western blot analysis

The BLA tissue samples of mice were added to radioimmunoprecipitation assay (RIPA) lysis, oscillated on a swirl instrument, and centrifuged at 4°C at 25764×***g*** with the supernatant collected. The protein concentration was then quantified using a bicinchoninic acid (BCA) protein assay kit. Next, 25 μg protein was dissolved in 2 × sodium dodecyl sulfate (SDS) loading buffer, and boiled at 100°C for 5 min. The protein was separated by 10% SDS/polyacrylamide gel electrophoresis (SDS/PAGE) gel and transferred onto polyvinylidene fluoride (PVDF) membranes using the wet transfer method. Membrane blockade was performed using 5% skim milk powder for 1 h at room temperature followed by incubation with diluted primary mouse monoclonal antibodies (Abcam Inc., Cambridge, MA, U.S.A.) against brain-derived neurotrophic factor (BDNF; ab108319, 1:1000), cAMP-responsive element-binding protein (CREB; ab178322, 1:500) and GAPDH (ab8245, 1:1000). The membrane was then washed three times using Tris-buffered saline Tween-20 (TBST) buffer (5 min for each), and incubated with the horseradish peroxidase (HRP)-conjugated secondary antibody for 1 h. After six TBST washes, the enhanced chemiluminescence (ECL) reagent kit (BB-3501, Amersham Pharmacia Biotech, Little Chalfont, U.K.) was employed to visualize the results under a Bio-Rad image analysis system (Bio-Rad, Hercules, CA, U.S.A.) and the Quantity One v4.6.2 software was performed to analyze the gray value.

### Statistical analysis

The SPSS 21.0 software (IBM Corp., Armonk, NY, U.S.A.) was used for statistical analysis. Measurement data were expressed as mean ± standard deviation. The normal distribution and homogeneity of variances were examined. Following the normal distribution and homogeneity of variances, comparisons between two groups were performed using unpaired *t*-test, and comparisons among multiple groups were assessed by one-way analysis of variance (ANOVA) or repeated measures ANOVA. Post-hoc test was conducted for pairwise comparison. If the data failed to meet the criteria of normal distribution or homogeneity of variances, a rank sum test was performed. A *P* value of <0.05 was considered to be statistically significant.

## Results

### Serum level of TH is increased and that of estrogen and progestogen are reduced in PPD patients and mice

**Figure 1:**
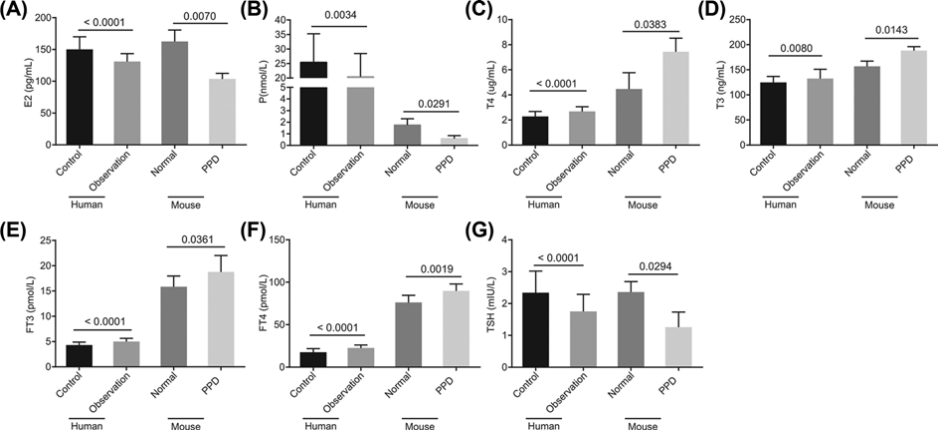
PPD leads to an increased TH level and decreased estrogen and progestogen (**A**–**G**) Serum levels of E2, P, T3, T4, FT3, FT4 and TSH in patients and mice detected by ECLIA; measurement data were expressed as mean ± standard deviation and analyzed by unpaired *t*-test; the control group: *n*=60; the observation group: *n*=58; the normal group: *n*=3; the PPD group: *n*=3. E2, estradiol; P, progesterone; T3, triiodothyronine; T4, thyroxine.

Primarily, we determined the serum levels of TH and estrogen and progestogen in PPD patients and mice. The results revealed that compared with the observation and PPD groups, serum levels of E2, P, and TSH were increased, while those of T3, T4, FT3 and FT4 were decreased in the control and normal groups (*P*<0.05; Figure 1). These results demonstrated that TH serum levels were elevated, while the serum levels of estrogen and progestogen were reduced in PPD patients and mice.

### Reduced TH combined with elevated estrogen and progestogen promotes the body weight and activities of PPD mice

**Figure 2:**
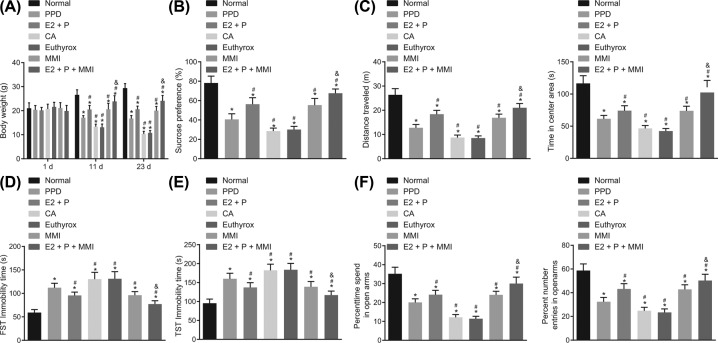
Reduction of TH combined with elevated estrogen and progestogen improves body weight and activities of PPD mice (**A**) The body weight of mice at each time point; (**B**) the consumption of sucrose solution of mice by SPT; (**C**) horizontal and vertical movement of mice by OFT; (**D**) the immobility time of mice in forced swimming by FST; (**E**) the immobility time of mice in tail suspension by TST; (**F**) the times of mice entering the open arms and the time spent in the open arms by EPM; *P<0.05 vs. the normal group; ^#^*P*<0.05 vs. the PPD group; ^&^*P*<0.05 vs. the E2 + P group; measurement data were expressed as mean ± standard deviation and analyzed by one-way ANOVA; data in Panel A were analyzed using repeated measures ANOVA; *n*=8. E2, estradiol; P, progesterone.

Next, the body weight and behavior of mice were analyzed following drug administration. The results indicated that, in comparison with the normal mice, the body weight of mice exhibited a gradual decline in the remaining groups (*P*<0.05). As shown in Figure 2A, compared with the PPD group, the body weight of mice administrated with E2 + P, MMI, or E2 + P + MMI was increased, yet decreased in mice administrated with CA or Euthyrox (*P*<0.05). When compared with the mice administrated with E2 + P or MMI, a higher weight was observed in mice administrated with E2 + P + MMI (*P*<0.05). In comparison with the normal mice, the consumption of sucrose solution, horizontal and vertical metamorphopsia scores of mice in the other groups were reduced (*P*<0.05). Mice administrated with E2 + P, MMI or E2 + P + MMI exhibited increased consumption of sucrose solution, horizontal and vertical metamorphopsia scores, as compared with PPD mice (*P*<0.05), while mice administrated with CA or Euthyrox presented an opposite trend (*P*<0.05). Additionally, mice administrated with E2 + P + MMI displayed higher consumption of sucrose solution, horizontal and vertical activities than those administrated with E2 + P (Figure 2B,C). Compared with the normal mice, the immobility time of the mice during forced swimming and tail suspension was prolonged in the remaining groups (*P*<0.05). Compared with PPD mice, the immobility time of swimming and tail suspension in mice administrated with E2 + P, MMIor E2 + P + MMI was shortened (*P*<0.05), while a longer period was detected in mice administrated with CA or Euthyrox (*P*<0.05). In addition, when compared with mice administrated with E2 + P, mice administrated with E2 + P + MMI exhibited a far shorter immobility time of swimming and tail suspension (*P*<0.05; Figure 2D,E). In comparison with the normal mice, the times of mice entering the open arms and the time spent in the open arms in the remaining groups were significantly reduced (*P*<0.05). Mice administrated with E2 + P, MMI or E2 + P + MMI exhibited a marked increase in the number of times of mice entering the open arms and the time spent in the open arms when compared with the PPD mice, while contrasting tendencies were observed in the mice administrated with CA or Euthyrox (*P*<0.05). Moreover, mice administrated with E2 + P + MMI displayed a significantly greater number of times entering the open arms and time spent in the open arms than mice administrated with E2 + P (*P*<0.05; Figure 2F). Taken together, the results obtained indicated that reduced TH combined with increased estrogen and progestogen can increase the body weight and activities of PPD mice in a more effective manner.

### Neuron cell morphology in BLA of PPD mice

**Figure 3:**
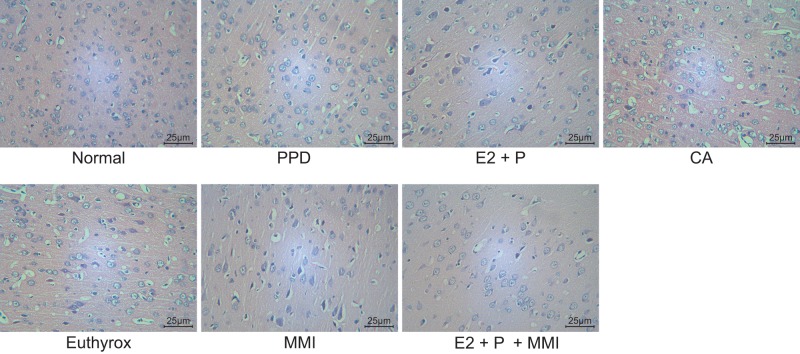
Reduction of TH combined with increased estrogen and progestogen enhances cell morphology of PPD mice (×400) The experiment was repeated three times independently; E2, estradiol; P, progesterone.

In order to investigate the effects of TH combined with estrogen and progestogen on neuron cell morphology, hematoxylin–eosin (HE) staining was conducted, the results of which revealed that mice in the normal mice showed increased neuron cells with complete structure, good morphology, plump cytoplasm and clear nucleus staining in BLA. In contrast, the PPD mice exhibited decreased neuron cells with enlarged gaps, more loose arrangement and unclear nucleus staining in BLA. Mice administrated with E2 + P or MMI had increased closely arranged neuron cells with narrowed gaps and clear nucleus staining in BLA as compared with PPD mice. Mice administrated with E2 + P + MMI had superior cell morphology to mice administrated with E2 + P. Neuron cells were significantly decreased in mice administrated with CA or Euthyrox, with the largest gaps and unclear nucleus staining (Figure 3).

### Synapse structure in BLA neurons of PPD mice

**Figure 4:**
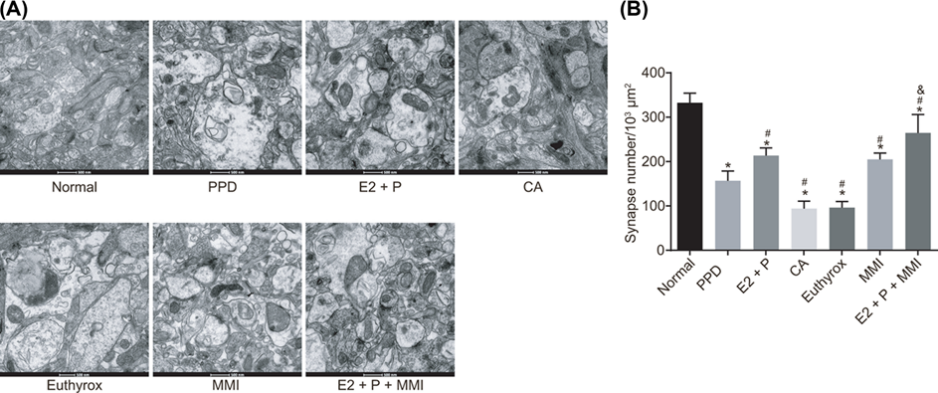
TH decline combined with restored estrogen and progestogen increases synapse density and improves synapse structure in BLA neurons of PPD mice (**A**) Images of synapse structure in BLA neurons of mice observed under TEM (×20000); (**B**) synapse density in BLA neurons of mice by TEM; *P<0.05 vs. the normal group; ^#^*P*<0.05 vs. the PPD group; ^&^*P*<0.05 vs. the E2 + P group; measurement data were expressed as mean ± standard deviation and analyzed by one-way ANOVA; the experiment was repeated three times independently; *n*=5. E2, estradiol; P, progesterone.

To explore the effects of TH combined with estrogen and progestogen on synapse density and structure in BLA neurons, TEM was employed to observe and analyze the synapse structure. The results obtained indicated that the synapse density in BLA neurons of PPD mice was decreased and the synapse structure was abnormal when compared with the normal mice. In comparison with the PPD mice, the synapse density in BLA neurons of mice administrated with E2 + P or MMI was enhanced, and the synapse structure improved, while the synapse density was reduced and the synapse structure displayed a clear abnormity in the mice administrated with CA or Euthyrox. Mice administrated with E2 + P + MMI had a higher synapse density and more obvious improvement than mice administrated with E2 + P (Figure 4). It can be concluded that the decrease in TH and increase in estrogen and progestogen can improve synapse density and structure in BLA neurons of PPD mice.

### Reduced TH combined with elevated estrogen and progestogen enhance the expression of BDNF and CREB in BLA of PPD mice

**Figure 5:**
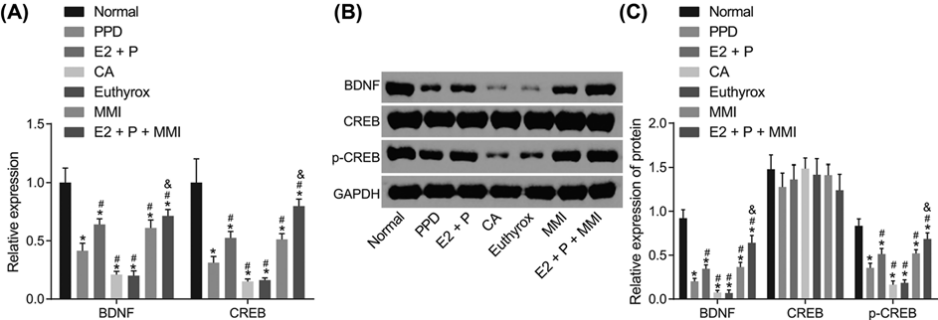
TH decline combined with restored estrogen and progestogen increases the expression of BDNF and CREB in BLA of PPD mice (**A**) The mRNA expression of BDNF and CREB in BLA of mice by RT-qPCR; (**B**) gray value of BDNF, CREB and p-CREB protein bands in BLA of mice by Western blot analysis; (**C**) protein levels of BDNF and CREB and the extent of CREB phosphorylation in BLA of mice; *P<0.05 vs. the normal group; ^#^*P*<0.05 vs. the PPD group; ^&^*P*<0.05 vs. the E2 + P group; measurement data were expressed as mean ± standard deviation and analyzed by one-way ANOVA; the experiment was repeated three times independently; *n*=5. E2, estradiol; P, progesterone.

In order to elucidate the effects of the combined administration on BDNF and CREB in BLA of PPD mice, reverse transcription quantitative polymerase chain reaction (RT-qPCR) and Western blot analysis were employed to detect the expression of BDNF and CREB. As depicted in Figure 5, compared with the normal mice, the mRNA and protein level of BDNF, mRNA level of CREB and the extent of CREB phosphorylation were decreased in all the PPD mice (*P*<0.05). In comparison with the PPD mice, the mRNA and protein level of BDNF, mRNA level of CREB and the extent of CREB phosphorylation were all increased in the mice administrated with E2 + P or MMI (*P*<0.05), while diminished levels were detected in the mice administrated with CA or Euthyrox (*P*<0.05). Mice administrated with E2 + P + MMI displayed increased mRNA and protein levels of BDNF, mRNA level of CREB and the extent of CREB phosphorylation, as compared with mice administrated with E2 + P (*P*<0.05). No significant difference was detected in the CREB protein levels in each group (*P*>0.05). These findings demonstrated that TH decline combined with increased estrogen and progestogen could effectively enhance the expression of BDNF and CREB in the BLA of PPD mice instead of single administration.

## Discussion

PPD is a widespread and depressive disorder seriously affecting the lives of new mothers and their families [23]. Hormone therapy is widely considered as a critical pharmacological option in the prevention or treatment of PPD [24]. Recent evidence has demonstrated that either estrogen or progestogen may be of prophylactic and/or therapeutic value in PPD [25]. In the current study, the results indicated that the combined administration of reduced TH with elevated estrogen and progestogen is more efficient to alleviate the symptoms of PPD than only single administration.

Women suffering from PPD appear to be more prone to postpartum hormonal changes than those who do not suffer from this form of depression [26]. Among new mothers, postpartum thyroid dysfunction is a common phenomenon [27]. Accordingly, one finding from our study was that PPD patients and mice presented with a higher serum level of TH (T3, T4, FT3 and FT4) and lower levels of TSH, estrogen and progestogen (E2 and P). Serum TH has predictive value in certain cerebrovascular diseases, which shows a neuroprotective role in the central nervous system through genomic and nongenomic actions under ischemic conditions [28]. Reports have presented evidence revealing a distinct positive association between serum thyroid-related hormone T3 in depressive patients and disease course [29]. Women with depression have a much lower serum TSH level than the healthy women, suggesting depression causes thyroid dysfunction [30]. Suppressed serum TSH and elevated serum T3 and T4 concentration lead to maternal overt hyperthyroidism, which may result in depressive symptomatology [31,32]. The risk of PPD has been directly linked with serum TSH, free thyroid hormones and thyroid autoantibodies [14] Additionally, estrogen has been demonstrated to engage in normal brain function since it can exert neurotrophic and neuroprotective actions [10]. As a female sex hormone, P has been widely documented to trigger secretion changes in the lining of the uterus pivotal for the successful implantation of a fertilized egg [33]. Furthermore, the changes of serum levels of E2 and P could be blamed for the emergency of PPD since the serum levels of E2 and P in the PPD group were significantly lower than those in the control group [34].

Consistently, another important finding from the present study was that the combined administration of reduced TH and elevated estrogen and progestogen can improve the body weight and activities of PPD mice. TH represents a crucial factor involved in the development of the brain, and even mild TH alteration in utero can initiate neuropsychological defects in children in spite of normal thyroid status at birth [35]. High levels of plasma FT4 can accelerate cognitive impairment, and increased serum T4 and low TSH have been shown to increase the risk of Alzheimer’s disease (AD) in individuals [36]. Moreover, PPD rats continually injected with high serum levels of E2 exhibit decreased immobility and increased struggling and swimming behaviors, as well as greater area crossed in the OFT [37]. In encephalomyelitis mice, P could attenuate clinical severity, retard neuronal dysfunction, and elevate axonal counts, while reducing the alterations of growth-associated proteins [12]. Moreover, we found that reduced TH and elevated estrogen and progestogen increased the expression of BDNF and CREB in BLA. The neuroplastic pathway, including CREB, BDNF and its receptor (neurotrophic tyrosine kinase receptor, type 2 [NTRK2]), has a key role to play in the adaptation of the brain to stress, with the variations of these genes highlighted as potential depression risk factors [38]. The PPD has been attributed to the rapid perinatal change in reproductive hormones [39]. Galanin (GAL), as an estrogen-inducible neuropeptide has been linked to depression, with GAL receptor1-siRNA injection reported to reverse the down-regulation of CREB-BDNF and 5-HT levels in the prefrontal cortex of PPD rats [40]. Similarly, enhanced thyroid peroxidase antibody elevates risk of PPD by diminishing BDNF and 5-HT levels in the prefrontal cortex of mice [41]. The deletion of CREB-regulated transcription coactivator 1 (CRTC1) has the capacity to induce impulsive aggressiveness, behavioral despair, psychomotor retardation, increased emotional response to stressful events, anhedonia, and depression-related behaviors [42]. After delivery, the ablation of estrogen is observed to reduce the hippocampal BDNF levels, which acts to impair hippocampal neurogenesis and initiates depression- and anxiety-like behaviors in mice [43]. Gonzalez et al. concluded that P confers neuroprotection in spinal cord trauma by up-regulating BDNF in motoneurons [44]. BDNF possesses the ability to reverse inhibition of granule cell neurite extension induced by hexabromocyclododecane (HBCD) in the presence of T3 [45].

The present study suggested the potential role of the three commonly studied hormones in relation to PPD, and revealed neuroprotective and antidepressant effects of the combined administration of E2, P, and MMI, which reduced TH and increased estrogen and progestogen via CREB-BDNF system, in the progression of PPD. Further studies exploring more factors would be beneficial for the treatment of PPD.

## Abbreviations

ANOVA: analysis of variance
BCA: bicinchoninic acid
BDNF: brain-derived neurotrophic factor
BLA: basolateral amygdala
CA: contraceptives
CRE: cAMP-responsive element-binding protein
ECL: enhanced chemiluminescence
ECLIA: electrochemiluminescence immunoassay
EPDS: Edinburgh Postnatal Depression Scale
EPM: elevated plus-maze
FST: forced swimming test
FT3: free triiodothyronine
FT4: free thyroxine
GAPDH: glyceraldehyde-3-phosphate dehydrogenase
HE: hematoxylin–eosin
MMI: methimazole
OFT: open field test
PPD: postpartum depression
RIPA: radioimmunoprecipitation assay
RT-qPCR: reverse transcription quantitative polymerase chain reaction
SPT: sucrose preference test
TBST: Tris-buffered saline Tween-20
TEM: transmission electron microscope
TH: thyroid hormone
TSH: thyroid-stimulating hormone
TST: tail suspension test
